# Supplementary figures and images for: The Desmin Mutation DES-c.735G>C Causes Severe Restrictive Cardiomyopathy by Inducing In-Frame Skipping of Exon-3
Source: Biomedicines. 2021 Oct 5;9(10):1400. doi: 10.3390/biomedicines9101400 (PMC8533191; doi:10.3390/biomedicines9101400)

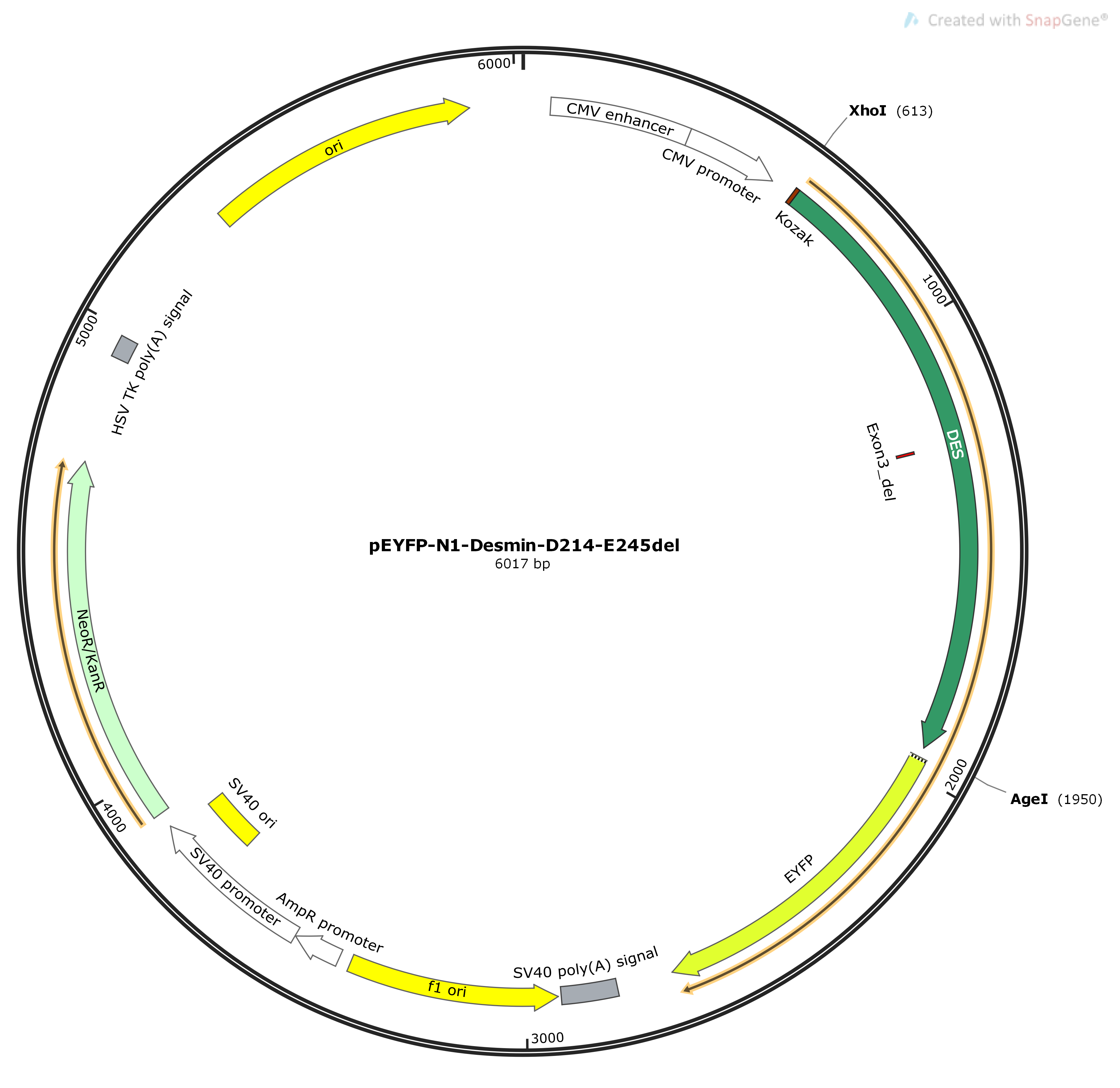

Supplement: Supplementary file 1 [file biomedicines-09-01400-s001.zip › biomedicines-1399677-supplementary-10.5/pEYFP-N1-Desmin-D214-E245del Map.png]

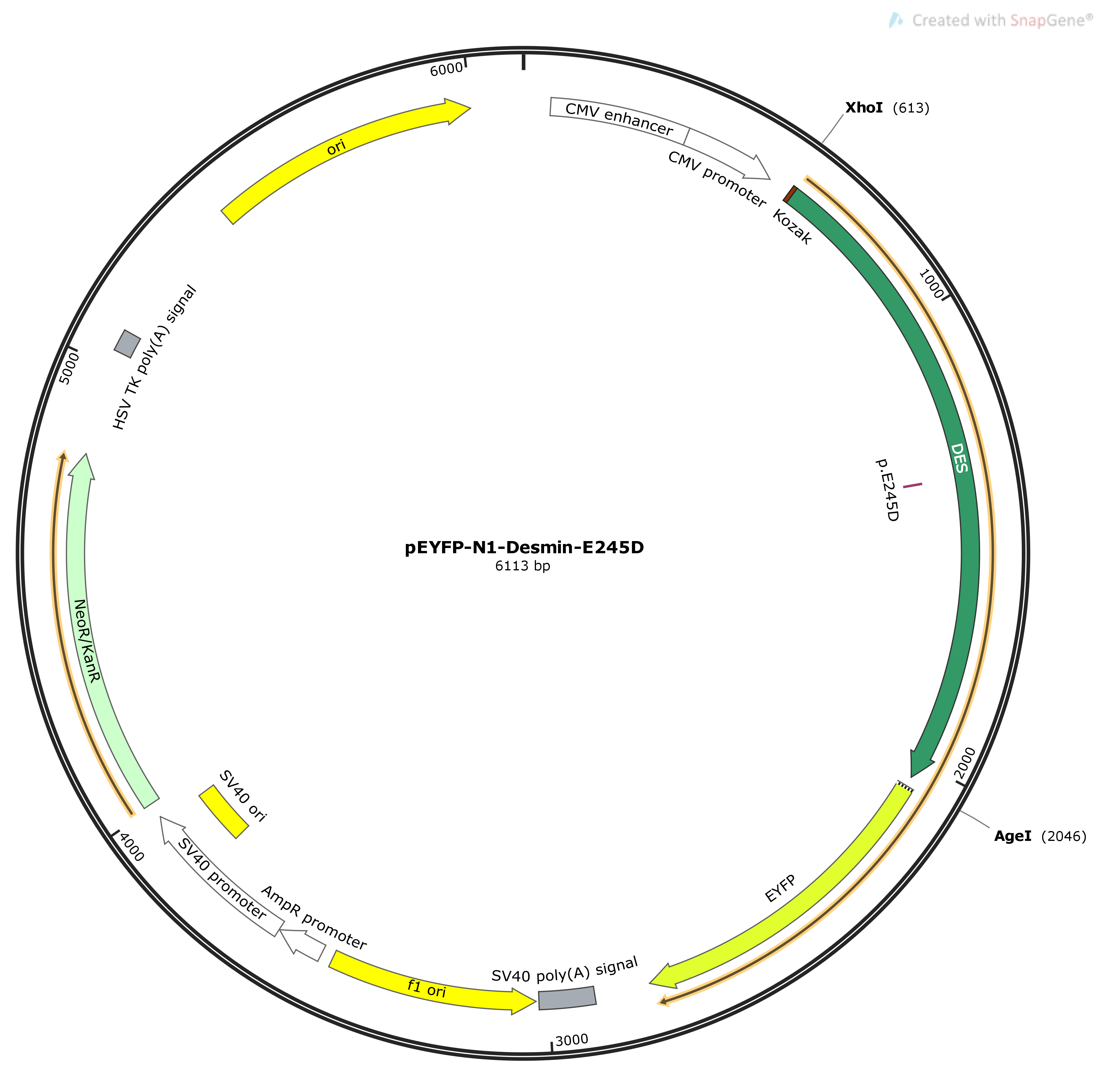

Supplement: Supplementary file 1 [file biomedicines-09-01400-s001.zip › biomedicines-1399677-supplementary-10.5/pEYFP-N1-Desmin-E245D Map.png]

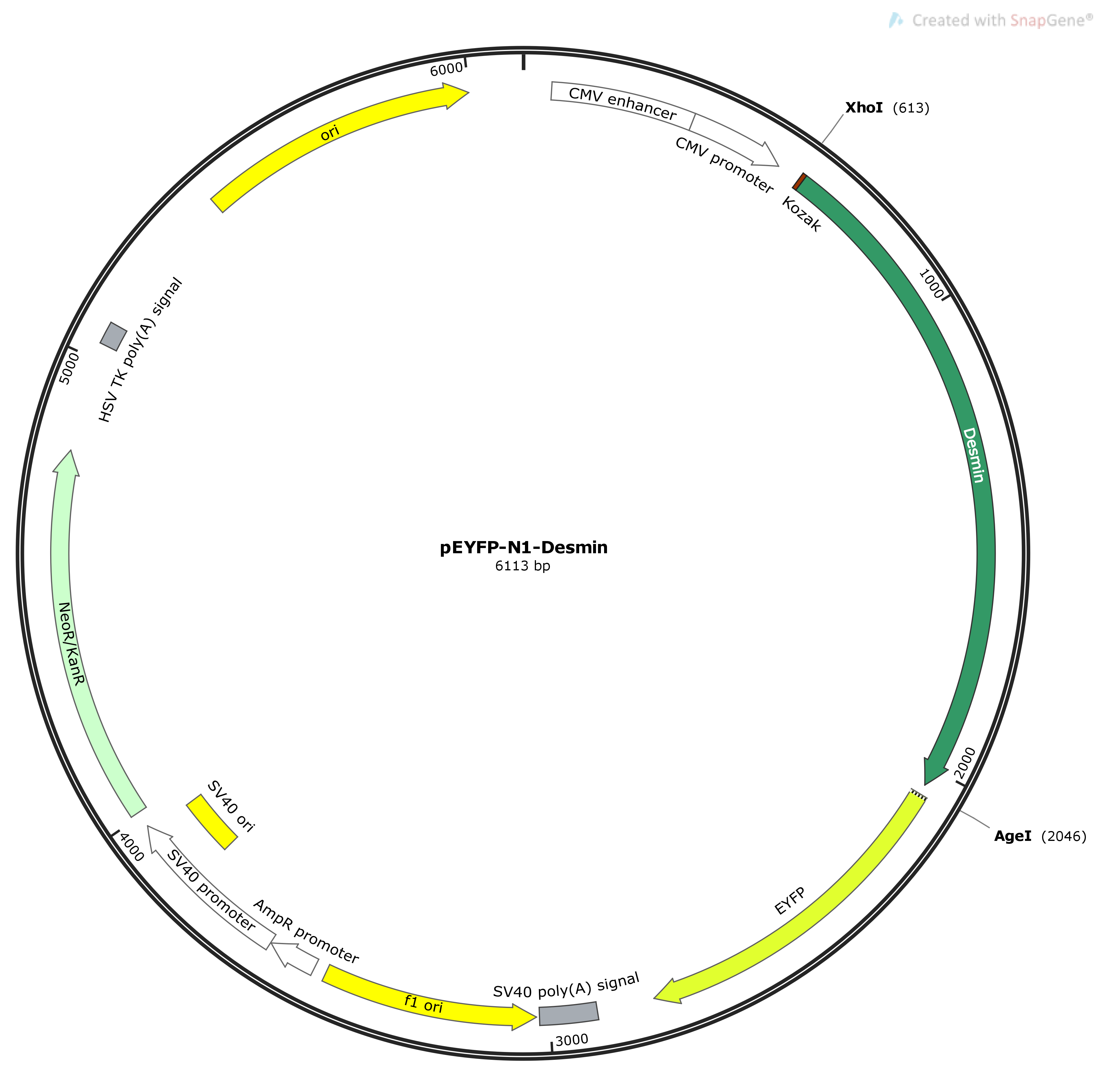

Supplement: Supplementary file 1 [file biomedicines-09-01400-s001.zip › biomedicines-1399677-supplementary-10.5/pEYFP-N1-Desmin-WT Map.png]
